# Superior Mesenteric Artery Syndrome Across Adulthood: A Retrospective Clinicoradiological Case Series

**DOI:** 10.3390/medicina62091812

**Published:** 2026-09-20

**Authors:** Alina Cristiana Venter, Gineta Andreescu, Oana Alexandra Isac, Timea Claudia Ghitea, Lavinia Florica Marcut, Corina Suteu, Cristian Marius Daina

**Affiliations:** 1 Department of Morphological Disciplines, Faculty of Medicine and Pharmacy, University of Oradea, 410073 Oradea, Romania; aventer@uoradea.ro (A.C.V.); gandreescu@uoradea.ro (G.A.); 2 Emergency County Hospital Bihor, 410169 Oradea, Romania; pavaleanuoanaalexandra@gmail.com; 3 Pharmacy Department, Faculty of Medicine and Pharmacy, University of Oradea, 1 December Sq., 410081 Oradea, Romania; 4 Department of Surgical Disciplines, Faculty of Medicine and Pharmacy, University of Oradea, 410073 Oradea, Romania; lmarcut@uoradea.ro; 5 Department of Psycho-Neurosciences and Recovery, Faculty of Medicine and Pharmacy, University of Oradea, 1 December Sq., 410081 Oradea, Romania; corina.suteu@didactic.uoradea.ro (C.S.); cdaina@uoradea.ro (C.M.D.)

**Keywords:** superior mesenteric artery syndrome, Wilkie syndrome, duodenal obstruction, adult case series, computed tomography, aortomesenteric angle, vascular compression findings, Nutcracker phenomenon, May–Thurner anatomy, gastric outlet obstruction

## Abstract

*Background and Objectives*: Superior mesenteric artery syndrome (SMAS) is an uncommon cause of proximal duodenal obstruction that requires clinicoradiological correlation. *Materials and Methods*: We retrospectively reviewed 13 patients with SMAS identified at the Clinical County Emergency Hospital Bihor, Romania, during 2019–2024. Clinical records and contrast-enhanced computer tomography (CT) examinations were reviewed descriptively. *Results*: The cohort included 13 adults aged 19–85 years (mean 45.5 ± 17.8; median 43 years). Abdominal pain was reported in 12/13 patients (92.3%), vomiting or emetic symptoms in 6/13 (46.2%), feeding difficulty or appetite disturbance in 5/13 (38.5%), and documented weight loss in 3/13 (23.1%). CT measurements were available for the aortomesenteric angle in 7/13 patients (range, 10–25°) and for the aortomesenteric distance in 8/13 (range, 3–8 mm); direct third-part duodenal compression and proximal dilatation were interpreted together with compatible symptoms. Imaging showed Nutcracker-type left renal vein compression in 3/13 patients (23.1%) and possible May–Thurner anatomy in 1/13 (7.7%); these findings were not adjudicated as clinical syndromes without corresponding manifestations. Based on the management labels, 9/13 patients (69.2%) underwent surgery at some point and 4/13 (30.8%) received medical therapy only. Standardized follow-up and procedure-level outcome data were not available for all patients. *Conclusions*: This small, retrospective series is descriptive and cannot estimate prevalence, diagnostic delay, or comparative treatment effectiveness. It illustrates heterogeneous adult presentations and supports interpreting aortomesenteric measurements together with compatible symptoms and evidence of duodenal obstruction.

## 1. Introduction

Superior mesenteric artery syndrome (SMAS), or Wilkie syndrome, is an uncommon cause of proximal intestinal obstruction caused by compression of the third portion of the duodenum between the abdominal aorta and the superior mesenteric artery (SMA). Symptoms may be acute, subacute, or chronic and can include postprandial pain, vomiting, early satiety, feeding intolerance, and weight loss [1,2].

Narrowing of the aortomesenteric angle and distance is often associated with loss of the retroperitoneal fat cushion, but clinically relevant obstruction can occur across adulthood and is not defined by body habitus alone. Cachexia, malignancy, severe weight loss, spinal deformity or surgery, and individual anatomical configuration have all been described as possible contributors [3,4,5]. In this context, BMI and weight loss are relevant clinical variables, but they were not systematically available for every patient in the present series.

CT can demonstrate the vascular relationship, direct compression of the third duodenal portion, and proximal dilatation. However, reduced angle or distance values can also occur without symptomatic obstruction, so imaging measurements should not be interpreted in isolation [6,7,8,9]. Similarly, compression of the left renal vein or iliac vein should be described as an imaging phenomenon or anatomical finding unless compatible clinical manifestations support a clinical syndrome.

Most published evidence consists of case reports and small case series. The rarity of SMAS and the heterogeneity of its clinical presentation make descriptive clinicoradiological documentation useful, while the absence of a comparator group limits causal or comparative conclusions.

The objective of this study was to provide a descriptive characterization of clinical presentation, CT findings, associated vascular compression findings, management classification, and available short-term documentation in 13 retrospectively identified adult patients with SMAS at a single institution. We did not prespecify diagnostic delay, prevalence, or treatment-effectiveness endpoints.

## 2. Materials and Methods

### 2.1. Study Design and Patient Selection

This retrospective observational case series was conducted at the Emergency County Clinical Hospital Bihor, Oradea, Romania, and covered the period 2019–2024. Cases were identified from available hospital clinical records and CT imaging examinations using the recorded diagnosis of SMAS/Wilkie syndrome, followed by manual confirmation of clinical and imaging compatibility. The exact electronic search query and whether ICD coding, a radiology registry, discharge diagnoses, or a surgical registry were used as the initial retrieval source were not retained in the study materials; this is acknowledged as a potential source of selection bias. Patients were included when the record documented compatible symptoms together with CT evidence of narrowing of the aortomesenteric space and direct compression or functional obstruction at the third part of the duodenum. Commonly cited angle (<22–25°) and distance (<8–10 mm) thresholds were treated as supportive rather than sufficient. When numeric measurements were absent from the original report, patients could still meet the inclusion criteria through direct documented compression, proximal dilatation or stasis, and clinical compatibility. The final cohort comprised 13 adults aged 19–85 years.

### 2.2. Clinical Data Collection

Clinical information was retrospectively extracted from electronic medical records. We recorded age, presenting symptoms, symptom-pattern category, associated diseases, documented nutritional compromise, previous investigations when mentioned, management labels, and any available short-term clinical documentation. Acute presentation was defined for this reanalysis as symptoms developing over ≤7 days; subacute as >7 days to 3 months; chronic as >3 months; and chronic with acute exacerbation as a chronic symptom pattern with a superimposed acute worsening. These categories were assigned from the narrative record and were not based on a prospectively recorded symptom diary. Baseline weight, BMI, recent weight loss, albumin, and other nutritional indices were not systematically captured for all patients; therefore, only case-level descriptors such as cachexia, hypoproteinemia, edema, or documented weight loss are reported.

### 2.3. Imaging Assessment

All patients underwent contrast-enhanced multidetector CT with multiplanar reconstructions. The aortomesenteric angle was measured on a sagittal or oblique sagittal reconstruction as the angle between the longitudinal axes of the abdominal aorta and SMA. The aortomesenteric distance was the shortest distance between the anterior aortic wall and the posterior SMA wall at the level of the third portion of the duodenum. Measurements were extracted from the original clinical reports when available; the CT examinations were not uniformly re-measured for this study, and no blinded independent re-review was documented. The number, specialty, and experience of the original readers were not retained in the dataset. Consequently, “NR” in Table 1 means that a numeric value was not available in the source report, not that the patient lacked CT evidence of compression. CT review also considered direct D3 compression, proximal gastric/duodenal dilatation, stasis, and associated vascular compression findings. Complementary examinations were reviewed when present.

### 2.4. Treatment and Outcome Assessment

Treatment labels were transcribed from the clinical records and were not assigned by a study protocol. Conservative management could include nutritional support, oral or enteral feeding when documented, symptomatic medical therapy, correction of fluid/electrolyte abnormalities, positional measures, nasogastric decompression, and observation; the route, duration, and completeness of these components were not consistently available. Surgical treatment was recorded when the chart listed an operation, generally in the setting of acute obstruction or persistent symptoms/feeding intolerance. The source material did not consistently provide objective failure criteria for conservative therapy, the exact timing of escalation, or patient-level operative details; therefore, gastrojejunostomy and duodenojejunostomy could not be compared. Outcome assessment was limited to whatever short-term clinical documentation was present, with no standardized follow-up interval or validated outcome instrument.

### 2.5. Statistical Analysis

Because of the retrospective design, small sample, absent comparator group, and incomplete measurement-level data, analysis was descriptive only. Continuous variables are reported as mean ± standard deviation or median with interquartile range, as appropriate, and categorical variables as counts and percentages. No inferential comparisons, prevalence estimates, diagnostic-delay estimates, or causal treatment comparisons were performed. Management counts were audited against the patient-level labels in Table 1; “surgical treatment at any time” includes both surgery-only and medical-therapy-plus-surgery labels.

### 2.6. Ethical Considerations

This study was conducted in accordance with the principles of the Declaration of Helsinki. Clinical and imaging data were anonymized before analysis. The study was approved by the Ethical Committee of the Clinical County Emergency Hospital Bihor (approval code 22729; 13 July 2026), Oradea, Romania. Informed consent was obtained from all subjects involved in the study, as stated in the manuscript back matter.

## 3. Results

### 3.1. Patient Characteristics

A total of 13 adult patients with clinicoradiologically compatible SMAS were retrospectively identified. Age ranged from 19 to 85 years, with a mean of 45.5 ± 17.8 years and a median of 43 years (IQR 36–58 years). A complete sex distribution was not retained in the patient-level table and is therefore not reported. The age range supports an adult-spectrum description rather than an adolescent-specific analysis.

Clinical presentation was heterogeneous: three patients had an acute pattern, four a subacute pattern, five a chronic pattern, and one chronic symptoms with acute exacerbation. These categories were descriptive classifications based on chart narratives and should not be interpreted as validated prognostic strata.

**Figure 1:**
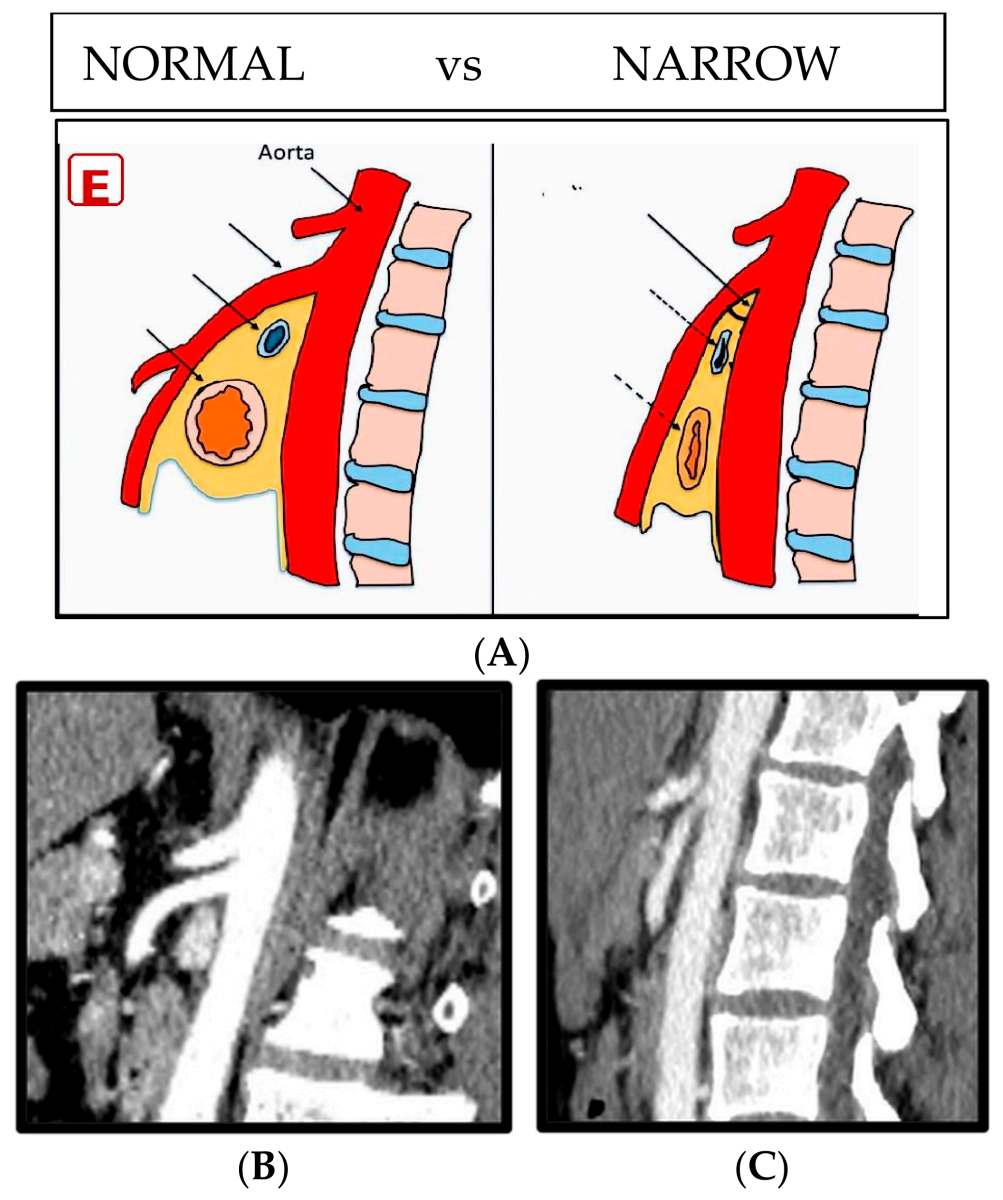

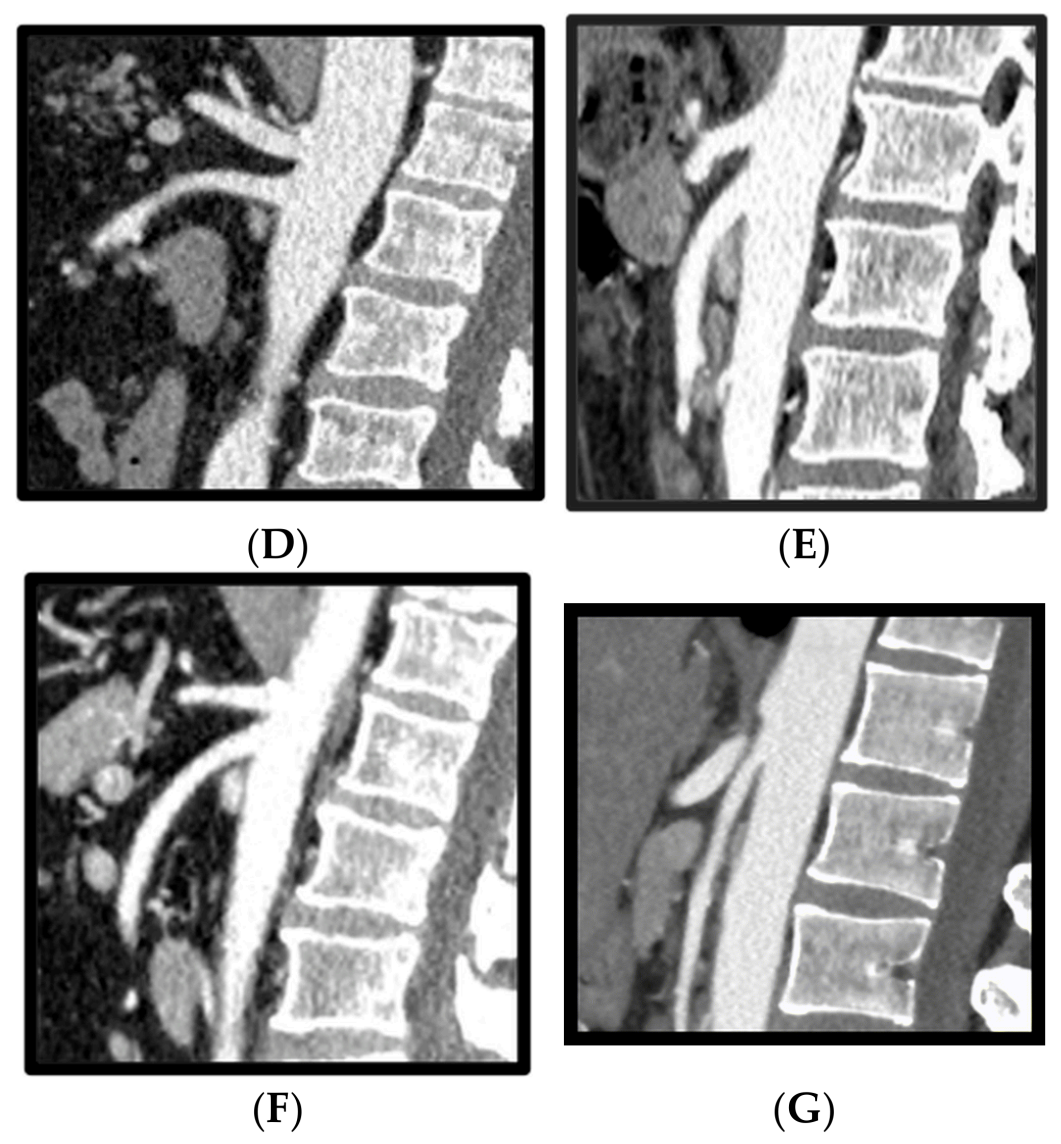
Representative CT images and schematic material related to the aortomesenteric vascular relationship. Panel labels (**A**–**G**) have been added to the image files. The panels are illustrative and are not a separate quantitative analysis.

Abdominal pain was documented in 12/13 patients (92.3%), vomiting or emetic symptoms in 6/13 (46.2%), feeding difficulty or appetite disturbance in 5/13 (38.5%), and documented weight loss in 3/13 (23.1%). Gastric stasis or dilatation of any degree was recorded in 8/13 (61.5%). Case-level nutritional compromise included malignancy-associated cachexia in two patients and hypoproteinemia/edema or documented weight loss in additional patients, but systematic BMI, weight, and laboratory nutritional data were unavailable. The phrase “Narrow Aorta Syndrome” in the original figure caption was unrelated to the present study and has been removed (Figure 1).

### 3.2. Clinical Presentation

The cohort included three acute, four subacute, five chronic, and one chronic-with-acute-exacerbation presentation. Abdominal pain was present in 12/13 (92.3%), vomiting or emetic symptoms in 6/13 (46.2%), feeding difficulty or appetite disturbance in 5/13 (38.5%), and documented weight loss in 3/13 (23.1%). These descriptive counts are based on the available chart documentation.

Gastric stasis or dilatation of any degree was recorded in 8/13 patients (61.5%). Marked stasis was described in four patients, delayed gastric emptying in one, and less pronounced stasis in three. The small series does not permit a severity comparison between presentations.

Acute presentations were described with more prominent distension and vomiting, whereas chronic and subacute presentations more often involved recurrent pain, dyspepsia, feeding intolerance, or intermittent vomiting. These are descriptive patterns from the records and were not tested statistically.

Nutcracker-type left renal vein compression was reported in 3/13 patients (23.1%), and one patient had possible May–Thurner anatomy on imaging. These findings are reported as imaging phenomena/anatomical findings because clinical syndrome criteria and related symptoms were not consistently adjudicated.

Other recorded conditions included malignancy-associated cachexia (n = 2), severe metabolic or nutritional derangements, chronic alcohol use, and a large hiatal hernia. The dataset did not contain standardized diagnostic-delay variables, so no quantitative conclusion about the duration of diagnostic delay, number of prior consultations, or number of previous investigations can be made.

**Figure 2:**
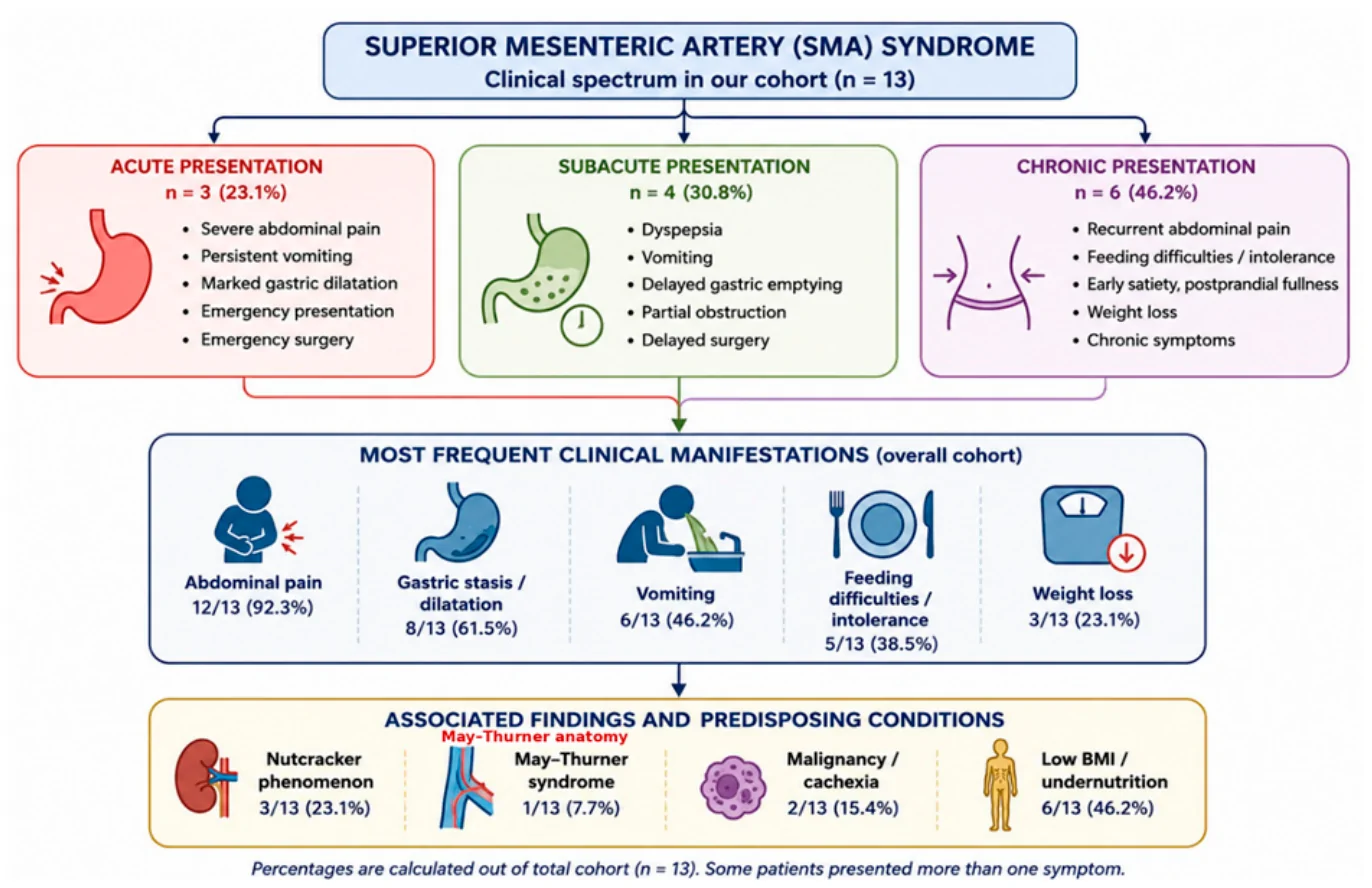
Descriptive clinical spectrum of SMAS in the study cohort (n = 13). Percentages are based on the total cohort; patients could have more than one symptom or associated finding. The label “May–Thurner anatomy” denotes an imaging finding and not a confirmed clinical syndrome.

All patients had clinical assessment and CT confirmation recorded, but the available records did not permit a standardized reconstruction of diagnostic pathways. Figure 2 is therefore descriptive and should not be read as evidence that all patients underwent the same sequence or duration of prior investigations (Table 2).

### 3.3. Imaging Characteristics

Contrast-enhanced CT with multiplanar reconstructions was performed in all 13 patients. A numeric aortomesenteric angle was available for 7/13 patients (range, 10–25°) and a numeric aortomesenteric distance for 8/13 (range, 3–8 mm). The remaining measurements were not available in the original reports and are marked NR in Table 1. Direct third-part duodenal compression, proximal dilatation or stasis, and compatible symptoms were considered together with the measurements.

The lowest recorded angle and distance values occurred in some patients with acute obstruction, but the dataset was too small and incomplete for an inferential comparison. This observation is presented descriptively and should not be interpreted as evidence that the absolute vascular measurements correlate with clinical severity.

Gastric and proximal duodenal dilatation varied across the cohort. Marked dilatation was described in several acute presentations, whereas some chronic presentations had less pronounced dilatation despite persistent symptoms. These observations support using functional evidence of obstruction and clinical compatibility alongside vascular measurements.

CT demonstrated narrowing at the third portion of the duodenum in the records supporting the diagnosis. The available material did not allow a blinded independent re-read or a formal reader-agreement analysis.

Imaging showed Nutcracker-type left renal vein compression in three patients and possible May–Thurner anatomy in one patient. Because compatible clinical manifestations were not consistently documented, these findings are not labeled as confirmed clinical syndromes.

Two patients had malignancy-associated cachexia with severe systemic disease. These case-level findings are clinically relevant but cannot be used to estimate the contribution of nutritional depletion in the wider SMAS population.

**Figure 3:**
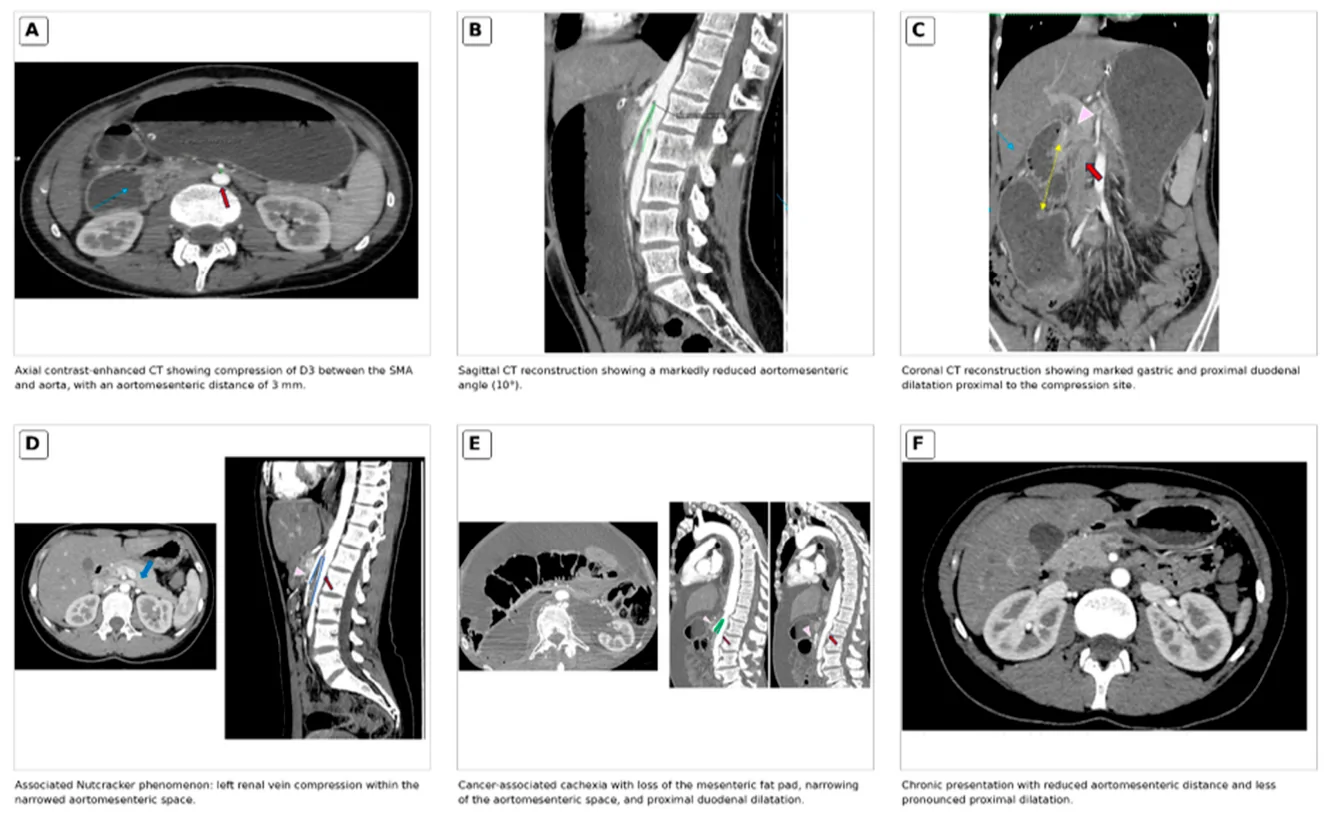
Representative imaging characteristics of superior mesenteric artery syndrome. (**A**) Axial contrast-enhanced CT demonstrating compression of the third portion of the duodenum between the superior mesenteric artery and the aorta, with an aortomesenteric distance of 3 mm. (**B**) Sagittal CT reconstruction showing a markedly reduced aortomesenteric angle of 10°. (**C**) Coronal CT reconstruction demonstrating marked gastric and proximal duodenal dilatation proximal to the site of compression. (**D**) Associated Nutcracker phenomenon, with compression of the left renal vein within the narrowed aortomesenteric space. (**E**) SMAS associated with cancer-related cachexia, demonstrating loss of the mesenteric fat pad, narrowing of the aortomesenteric space, and proximal duodenal dilatation. (**F**) Chronic presentation with reduced aortomesenteric distance and less pronounced proximal dilatation. Colored arrows, arrowheads, and measurement lines identify the relevant anatomical structures and imaging findings: in panel (**A**), define the cyan and red arrows and the green distance marker; in panel (**B**), the green lines indicate the measured aortomesenteric angle; in panel (**C**), define the cyan arrow, yellow double-headed arrow, pink arrowhead, and red arrow; in panel (**D**), define the blue and red arrows, blue measurement lines, and pink arrowhead; and in panel (**E**), define the green and red arrows and pink arrowhead.

Overall, the imaging findings were heterogeneous. The most defensible descriptive conclusion is that compatible symptoms, direct D3 compression, and proximal obstruction/stasis provided contextual support for SMAS when quantitative angle or distance measurements were missing (Figure 3).

### 3.4. Associated Vascular Compression Syndromes

Because SMAS involves the aortomesenteric compartment, the CT records were also reviewed for associated vascular compression findings. The results are reported as imaging observations and were not intended to diagnose Nutcracker or May–Thurner clinical syndromes without compatible manifestations.

Nutcracker-type left renal vein compression was documented in 3/13 patients (23.1%). This finding should be distinguished from Nutcracker syndrome, which requires appropriate clinical correlation.

One patient (1/13, 7.7%) had imaging features compatible with May–Thurner anatomy. The available records did not establish a symptomatic May–Thurner syndrome.

These observations may justify careful review of adjacent vessels when SMAS is suspected, but this selected 13-patient series cannot estimate the prevalence of associated compression disorders or establish a broader disease spectrum.

Among the remaining patients, no additional vascular compression finding was recorded. Other associated conditions included cachexia, severe malnutrition, chronic alcohol use, hiatal hernia, and unspecified systemic disease (Table 3).

### 3.5. Management and Outcomes

Treatment labels were heterogeneous and were not assigned using a prospective protocol. Based on Table 1, 9/13 patients (69.2%) underwent surgery at some point and 4/13 (30.8%) were listed as medical therapy only. Four patients were listed as surgery without preceding medical therapy and five as medical therapy + surgery. The source records did not consistently specify the exact timing of escalation, route/duration of nutritional therapy, or operative procedure.

Initial medical therapy, when documented, included nutritional support, symptomatic treatment, correction of fluid/electrolyte abnormalities, and clinical monitoring. The records did not consistently document nasogastric decompression, positional treatment, enteral access, duration of therapy, or standardized failure criteria.

The patient-level management labels reconcile to nine surgical cases and four medical-only cases. Among the 10 chronic or subacute presentations, one patient was listed as surgery without preceding medical therapy, five as medical therapy + surgery, and four as medical therapy only. These counts replace the inconsistent 7-versus-6 and 10-to-8 pathways reported previously.

The records indicate that both gastrojejunostomy and duodenojejunostomy were considered or listed in the manuscript materials, but patient-level procedure assignment and the criteria for choosing one operation over the other were not consistently available. Surgical and conservative groups therefore cannot be compared for effectiveness because treatment was determined by clinical severity and other indications.

Patients with malignancy-associated cachexia or substantial comorbidity were often managed non-operatively, illustrating potential confounding by indication. This pattern should not be interpreted as evidence that conservative treatment is superior or that surgery is ineffective.

**Figure 4:**
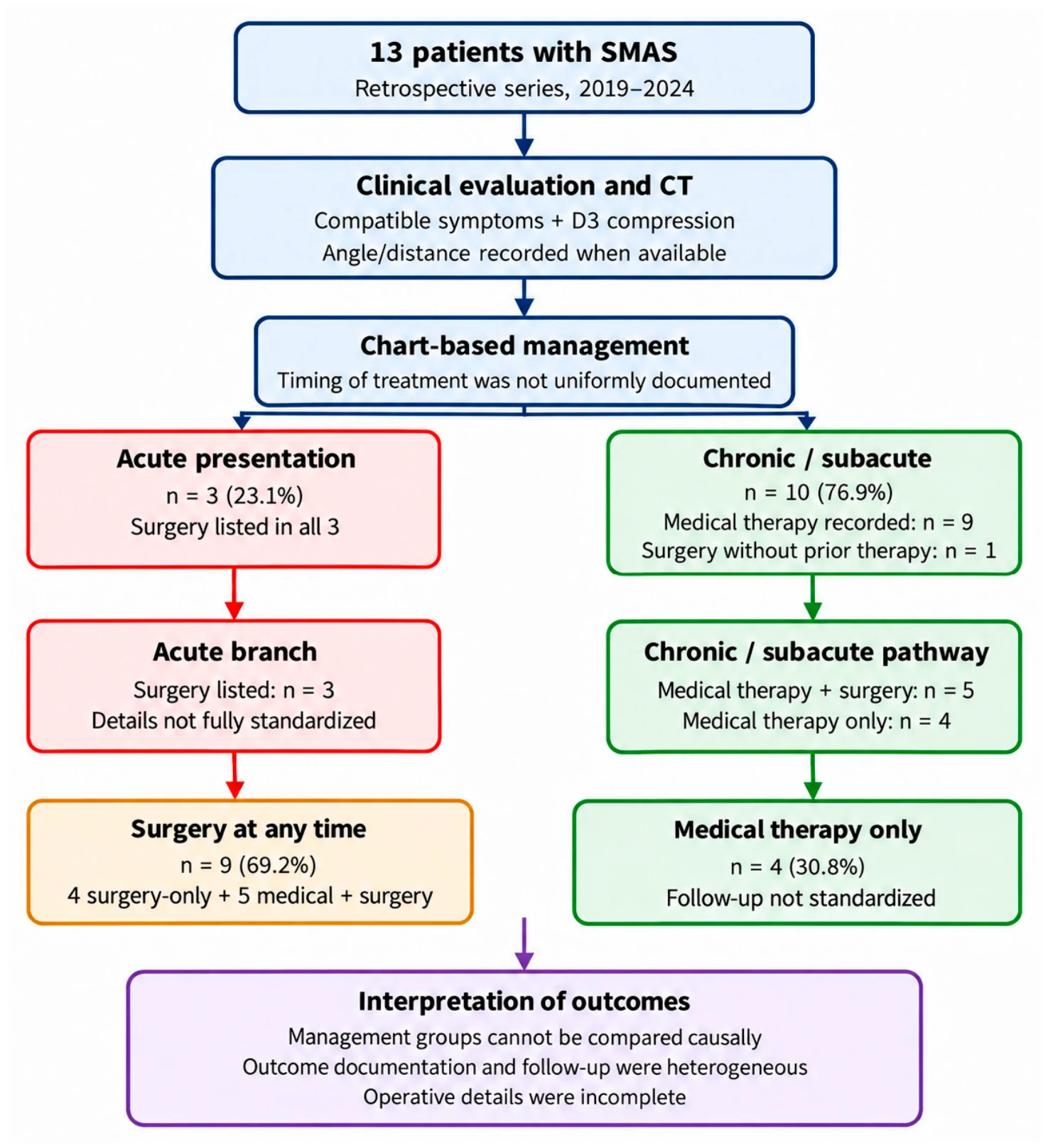
Corrected descriptive management pathway for the 13-patient cohort. Table 1 supports 9 patients with surgery at any point (69.2%) and 4 with medical therapy only (30.8%). Five patients were labeled medical therapy + surgery, four surgery-only, and four medical therapy-only. Outcome documentation and follow-up were heterogeneous; no causal comparison is implied.

Standardized follow-up was not available for all patients, and no validated definition or duration of “short-term improvement” was prespecified. The available notes therefore do not support a reliable numerical comparison of symptom resolution, nutritional recovery, or quality of life after surgery versus medical therapy (Figure 4 and Table 4).

## 4. Discussion

This retrospective series is descriptive. It includes 13 adults aged 19–85 years with heterogeneous acute, subacute, and chronic presentations. The most frequent documented manifestations were abdominal pain, vomiting or emetic symptoms, feeding difficulty, weight loss, and gastric stasis. CT measurements were incomplete, and diagnosis was supported by the combination of compatible symptoms, direct D3 compression, and proximal obstruction or stasis rather than by a numeric threshold alone.

This study did not collect standardized symptom duration, time to diagnosis, number of previous investigations, or prior diagnoses. Accordingly, it cannot demonstrate a “remarkable delay in diagnosis” or quantify a diagnostic gap. Published reports may discuss delayed recognition, but those observations should not be presented as measured findings of this cohort [10,11,12,13,14,15].

The age range illustrates that SMAS can be clinically relevant across adulthood; it does not support an adolescent-specific conclusion. The series also lacked systematic BMI, weight, and nutritional laboratory data, so statements about thin habitus, rapid somatic growth, or constitutional weight status should be attributed to prior literature or limited to the documented case-level findings [16,17,18,19].

The observed imaging heterogeneity is clinically plausible: some patients had marked proximal dilatation, whereas others had less pronounced dilatation despite compatible symptoms. In this series, however, the observations were not tested statistically, and the missing measurements preclude a quantitative relationship between angle/distance and severity [20,21,22,23,24].

Three of thirteen patients had Nutcracker-type imaging findings and one had possible May–Thurner anatomy. These findings may motivate careful radiological review, but a selected case series cannot estimate prevalence or establish that SMAS is part of a broader vascular-compression syndrome spectrum. The broader-spectrum interpretation is best regarded as a hypothesis for future work [25,26].

The presence of cachexia and severe systemic disease in some patients is consistent with published mechanisms involving loss of the mesenteric fat cushion, but the present study did not measure BMI, visceral fat, or weight trajectories systematically. The current data therefore support only a cautious clinical description [27,28,29].

Contrast-enhanced CT remains useful because it can demonstrate D3 compression, proximal dilatation, and adjacent vascular anatomy in one examination. The present records did not permit a reader-blinded re-review or formal diagnostic-accuracy analysis, so these statements are contextual rather than estimates of test performance [30].

Management should be individualized. In this series, nine patients had surgery listed at some point and four had medical therapy only, but the groups were defined by clinical severity and comorbidity. Confounding by indication prevents comparison of outcomes. Gastrojejunostomy and duodenojejunostomy are recognized operative options in the literature, yet this dataset did not reliably capture the procedure and indication for each patient [31].

The practical implication is not that early recognition was demonstrated to reduce investigations or improve outcomes but that compatible symptoms plus evidence of D3 obstruction should prompt consideration of SMAS. The present series cannot establish the duration of diagnostic pathways or the benefit of a particular diagnostic strategy [28].

Rather than proposing a conceptual shift, this case series supports a narrower hypothesis: SMAS may have heterogeneous adult presentations and may coexist with other imaging findings, but those observations require confirmation in larger, systematically phenotyped cohorts with standardized follow-up.

### 4.1. Clinical Implications

Clinically, persistent postprandial pain, recurrent vomiting, early satiety, feeding intolerance, or unexplained weight loss may justify review of the aortomesenteric relationship when more common causes have not explained the presentation. This is a potential clinical implication, not an outcome demonstrated by the present series.

### 4.2. Study Limitations

This study has important limitations. It was retrospective, single-center, and limited to 13 selected cases without a comparator group. The case-identification query, original CT reader characteristics, and standardized timing of clinical and imaging assessments were not retained. Angle measurements were available in only 7/13 patients and distance measurements in 8/13; “NR” reflected unavailable report values. BMI, weight, weight trajectory, and nutritional laboratory data were not systematic. Treatment labels did not provide complete information about route or duration of conservative therapy, objective failure criteria, operative procedure, or escalation timing. Follow-up was heterogeneous and not standardized, so short-term outcomes could not be quantified reliably. The series cannot estimate prevalence, diagnostic delay, or treatment effectiveness.

## 5. Conclusions

In this 13-patient retrospective series, SMAS showed heterogeneous clinical and imaging presentations across adulthood. The most defensible conclusion is that aortomesenteric angle and distance measurements should be interpreted together with compatible symptoms, direct third-part duodenal compression, and evidence of proximal obstruction or stasis. Imaging findings such as Nutcracker-type renal vein compression or possible May–Thurner anatomy should be described as phenomena or anatomical findings unless clinical criteria are documented. The management counts in this dataset indicate surgery at some point in nine patients and medical therapy only in four, but heterogeneous follow-up and confounding by indication preclude comparative outcome conclusions.

## Figures and Tables

**Table 1:** Patient-level clinical, imaging, and management characteristics of the retrospectively identified SMAS cohort (n = 13).

Patient	Age (years)	Aortomesenteric Angle (°)	Aortomesenteric Distance (mm)	Clinical Presentation	Main Clinical/Imaging Findings	Associated Conditions	Management
P1	19	NR	NR	Acute	Marked gastric stasis; occlusive syndrome	None reported	Surgery only (listed)
P2	22	12	5	Chronic	Abdominal pain; intermittent vomiting	None reported	Surgery only (listed)
P3	36	10	3–5	Chronic	Abdominal pain	Nutcracker phenomenon; possible May–Thurner anatomy	Medical therapy + surgery
P4	43	20	NR	Acute	Marked gastric stasis; epigastric pain; vomiting; weight loss	Chronic alcohol use	Surgery only (listed)
P5	32	22	8	Chronic	Abdominal pain; feeding difficulties; erythematous gastropathy	None reported	Medical therapy + surgery
P6	44	23	NR	Subacute	Gastric stasis; delayed gastric emptying; dyspeptic symptoms; emetic syndrome; pain	Nutcracker phenomenon	Medical therapy + surgery
P7	39	23	8	Acute	Marked gastric stasis; abdominal pain	None reported	Surgery only (listed)
P8	63	NR	6	Chronic with acute exacerbation	Pain; vomiting; weight loss; hypoproteinemia; edema; electrolyte disturbances	Low BMI/undernutrition documented in chart	Medical therapy + surgery
P9	41	NR	5	Chronic	Abdominal pain	Nutcracker phenomenon	Medical therapy + surgery
P10	59	NR	NR	Subacute	Gastric stasis; abdominal pain	Neoplastic disease with cachexia	Medical therapy only
P11	50	23	8	Chronic	Emetic syndrome; abdominal pain; feeding difficulties; erythematous gastropathy	Neoplastic disease with cachexia	Medical therapy only
P12	58	25	4–5	Subacute	Gastric stasis; abdominal pain; vomiting	Other associated disease, unspecified	Medical therapy only
P13	85	NR	NR	Subacute	Gastric stasis; subocclusive syndrome; abdominal pain	Large hiatal hernia involving approximately half of the stomach	Medical therapy only

Abbreviations: NR, numeric measurement not available in the source report; SMAS, superior mesenteric artery syndrome. Management labels were transcribed from the charts: 9 patients underwent surgery at some point (4 listed as surgery without preceding medical therapy and 5 listed as medical therapy + surgery), while 4 were listed as medical therapy only. Operative timing and exact procedure were not consistently documented.

**Table 2:** Clinical presentation and associated characteristics of patients with superior mesenteric artery syndrome (n = 13).

Clinical Variable	n (%)
Abdominal pain	12 (92.3)
Vomiting/emetic syndrome	6 (46.2)
Feeding difficulties/appetite disturbance	5 (38.5)
Weight loss documented	3 (23.1)
Gastric stasis (any degree)	8 (61.5)
Nutcracker-type imaging finding	3 (23.1)
Possible May–Thurner anatomy	1 (7.7)
Malignancy/cachexia	2 (15.4)
Acute presentation	3 (23.1)
Subacute presentation	4 (30.8)
Chronic presentation	5 (38.5)
Chronic with acute exacerbation	1 (7.7)

**Table 3:** Associated vascular compression findings identified on CT (n = 13). These are imaging findings; clinical syndromes were not adjudicated without compatible manifestations.

Associated Imaging Finding	n	%
Nutcracker-type left renal vein compression	3	23.1
Possible May–Thurner anatomy	1	7.7
No additional vascular compression recorded	9	69.2

**Table 4:** Reconciled management classification from patient-level chart labels (n = 13). Surgical treatment at any time includes surgery-only and medical-therapy-plus-surgery labels.

Management Classification from Table 1	n	%
Medical therapy only	4	30.8
Medical therapy + surgery	5	38.5
Surgery only (listed)	4	30.8
Surgical treatment at any time	9	69.2
Chronic/subacute presentations	10	76.9
Chronic/subacute with medical therapy recorded	9	69.2

## Data Availability

The original contributions presented in this study are included in this article. Further inquiries can be directed to the first author.

## Abbreviations

The following abbreviations are used in this manuscript:

BMI	Body mass index
CT	Computed tomography
NR	Not reported
SMA	Superior mesenteric artery
SMAS	Superior mesenteric artery syndrome
